# Tooth Decay: Genetic and Epigenetic Insights Driving the Development of Anti-Caries Vaccines

**DOI:** 10.3390/genes16080952

**Published:** 2025-08-12

**Authors:** Inès Bouaita, André Peixoto, Paulo Mascarenhas, Cristina Manso

**Affiliations:** 1 Egas Moniz Dental Clinic (EMDC), Instituto Universitário Egas Moniz, Campus Universitário, Quinta da Granja, Monte de Caparica, 2829-511 Almada, Portugal; 2 Centro de Investigação Interdisciplinar Egas Moniz (CiiEM), Instituto Universitário Egas Moniz, Campus Universitário, Quinta da Granja, Monte de Caparica, 2829-511 Almada, Portugal

**Keywords:** dental caries, anti-caries vaccine, genetic susceptibility, epigenetic mechanisms

## Abstract

Dental caries is now recognised as a multifactorial disease shaped by complex interactions among genetic, epigenetic, microbiological, environmental, and social factors. This narrative review synthesises recent findings on the influence of genetic and epigenetic factors on caries susceptibility, exploring implications for personalised prevention strategies, including novel vaccine approaches. Numerous gene polymorphisms in pathways related to enamel formation, saliva composition, immune response, and taste perception have been linked to increased caries risk, with some effects modulated by sex and tooth-specific factors. Early-life environmental exposures (diet, tobacco, and antibiotic use) have been demonstrated to further alter risk through epigenetic modifications such as DNA methylation, microRNA regulation, and histone changes. The recognition of this landscape of inherited and acquired vulnerabilities has given rise to interest in innovative preventive measures. In particular, anti-caries vaccines targeting *Streptococcus mutans* are being developed using protein subunits, DNA constructs, and even plant-based antigen production. Notwithstanding the challenges that still need to be overcome—chiefly the achievement of robust mucosal immunity, the assurance of safety, and the enhancement of production—these vaccines are proving to be a promising addition to traditional oral hygiene and fluoride measures. The integration of genetic and epigenetic insights with immunological advances has the potential to facilitate the development of more effective, personalised interventions to prevent dental caries.

## 1. Introduction

Dental caries is one of the most widespread diseases worldwide, affecting people of all ages [1]. It represents a significant public health concern with notable functional, social, and economic implications. Long seen as simply the result of poor hygiene or excessive sugar consumption, dental caries is now recognised as a multifactorial disease resulting from complex interactions between behavioural, environmental, microbiological, genetic, and epigenetic factors. This complexity explains why traditional prevention campaigns often have limited long-term effectiveness, highlighting the need for a more comprehensive and personalised approach [1].

Biologically, dental caries develops from the formation of bacterial biofilm on tooth surfaces, especially in the regular presence of fermentable sugars. This biofilm produces organic acids that lower the pH of the mouth, gradually leading to the demineralisation of the enamel and then the dentine. The frequency of sugar consumption, the quality of saliva, and the ability of the local immune system to defend itself strongly influence this process. This explains why some people develop dental caries quickly while others do not, even with similar habits. Differences in diet, the composition of the oral microbiota or the body’s resistance play a crucial role in this fragile balance [1].

Part of this variability between individuals can be explained by genetics. Some people are naturally more susceptible to dental caries due to variations in their genes, which influence tooth quality, saliva composition, taste perception or the oral immune response. Slight differences in the expression or regulation of specific genes can be enough to make someone more vulnerable to acid attacks or bacterial colonisation [2,3,4]. However, these genetic factors do not work alone; they constantly interact with the environment.

In this context, epigenetics emerges as a pivotal field of study. Therefore, it can be concluded that the environment can modulate the expression of specific genes without altering the deoxyribonucleic acid (DNA) sequence itself. A multitude of factors influence these epigenetic mechanisms, which in turn modify gene activity. Such factors include diet, stress, toxins, and microbiota. Recent studies have identified certain epigenetic signatures associated with increased vulnerability to dental caries. The potential for these markers to facilitate early identification of at-risk children is a promising avenue for future research. Thus, epigenetics links an individual’s genetic makeup with the environmental factors that shape their disease risk [5,6].

All these discoveries are paving the way for a new approach to prevention and treatment: personalised dentistry [6]. Clinicians are no longer limited to general advice. It is now possible to anticipate a patient’s caries vulnerability by considering their biological profile and lifestyle. Targeted strategies can be put in place, such as adapting diet, stimulating saliva production or strengthening local immune defences [7]. Among the emerging therapeutic strategies under investigation, the development of anti-caries vaccines stands out as a promising and actively explored approach [8]. By targeting the bacteria responsible for the disease, these vaccines could prevent *S. mutans* from adhering to teeth or reduce its virulence, providing long-lasting protection, particularly for the most exposed populations [8,9,10,11].

In short, dental caries should no longer be seen as a simple consequence of poor habits or excessive sugar consumption. It is the result of a continuous interplay between genetic predisposition, environmental exposures, and epigenetic regulation that orchestrate their interaction. It is in this complexity that we find the keys to better understanding, preventing, and treating this ancient yet ever-present disease. By embracing this more integrated vision, dentistry is gradually shifting toward a more preventive and personalised paradigm informed by emerging molecular evidence.

## 2. Genes Involved in Oral Health

**Figure 1:**
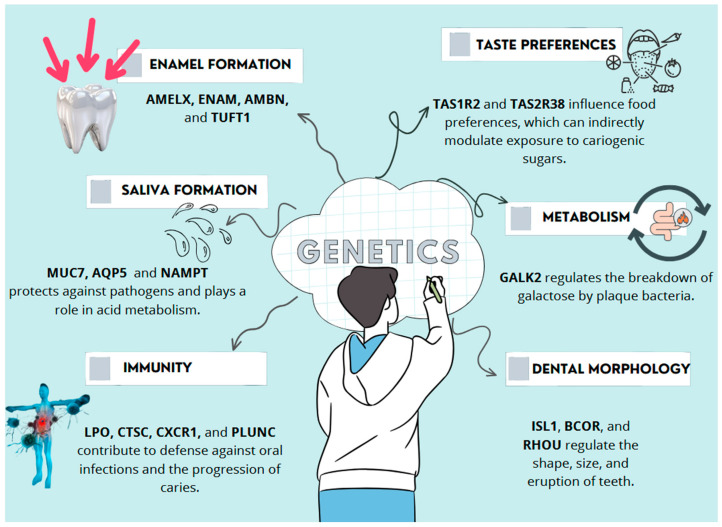
Representative mechanisms by which key genetic risk factors contribute to dental caries susceptibility [2,3].

Genetic factors contribute to dental caries susceptibility through multiple biological mechanisms. These include genes involved in enamel and dentine formation, salivary composition, immune defence, taste perception, and metabolic regulation. Their effects are not independent; variations in one pathway may influence or exacerbate vulnerabilities in another. For example, weaker enamel structure combined with reduced salivary buffering or a strong preference for sugary foods can significantly increase caries risk (Figure 1) [2,3].

### 2.1. Genes Involved in the Development of Dental Hard Tissues

The integrity of enamel and dentine is fundamental to resisting acid attacks and bacterial invasion. This resistance relies on the precise development and mineralisation of dental hard tissues, a process tightly regulated by multiple genes acting in a coordinated sequence, from early matrix formation to final tissue maturation.

During the initial stages of enamel formation, specialised epithelial cells known as ameloblasts secrete a complex protein matrix. Among the most essential genes at this stage are AMELX (Amelogenin), ENAM (Enamelin), and AMBN (Ameloblastin) [12,13]. These genes encode structural proteins that form the scaffold of the enamel matrix. When altered, they may cause hypomineralised or hypoplastic enamel, increasing susceptibility to demineralisation. For example, AMELX mutations can lead to thin and porous enamel, while changes in ENAM or AMBN affect matrix organisation and mineral stability [2,3,5,14,15,16,17]. Tuftelin 1 (TUFT1) also helps start enamel crystal formation and anchors the matrix to the dentine. Its variants can weaken enamel and raise caries risk [2,3,14,15,16,17,18].

As enamel development progresses, the organic components of the matrix must be removed to allow proper crystal growth. This is mediated by enzymes such as MMP20 (Matrix metalloproteinase 20) [2,5,15,16,18,19], KLK4 (Kallikrein-related peptidase 4), and TFIP11 (Tuftelin-interacting protein 11) [2,5,15,16,18]. These proteins break down excess matrix material, allowing the enamel to harden. Variants in these genes can delay or impair this critical maturation phase, leaving the enamel porous and fragile. Other enzymes, such as Matrix metalloproteinase 13 (MMP13) and Matrix metalloproteinase 16 (MMP16), are also involved in extracellular matrix remodelling and may influence enamel resilience [2,15,19].

Dentine development runs in parallel, supported by genes such as DSPP (Dentine sialophosphoprotein), a key player in dentine matrix mineralisation. Defects in DSPP compromise dentine structure and may indirectly weaken the enamel, promoting caries progression [17,20,21,22].

In addition to structural and enzymatic genes, several regulators modulate tooth development. The Polycystin-2 (Polycystic kidney disease 2 gene, PKD2) is involved in enamel–dentine signalling and tissue organisation, while ABCG2 (ATP-binding cassette sub-family G member 2) may help eliminate harmful substances during mineralisation [2]. The SCPP (Secretory calcium-binding phosphoprotein) gene family supports the formation of both enamel and dentine by stabilising mineral deposition [2].

Finally, upstream regulatory genes such as ESRRB (Oestrogen-related receptor beta), involved in ameloblast differentiation [16,17,18,20], and NEDD9 (Neural precursor cell expressed, developmentally down-regulated 9), influencing the migration of neural crest cells (precursors to ameloblasts and odontoblasts), contribute to overall tooth structure and resistance [2].

Altogether, these genetic factors shape the quality and durability of enamel and dentine. Variants in any of them can compromise tissue integrity and increase individual vulnerability to dental caries, supporting the rationale for future genetically based prevention strategies.

### 2.2. Genes Involved in Saliva Composition and Function

Saliva plays a pivotal role in oral homeostasis by lubricating tissues, neutralising acids, initiating digestion, and hosting a range of antimicrobial and remineralising agents. The composition and functionality of saliva are partly under genetic control, with several genes influencing its biochemical profile and, consequently, individual susceptibility to tooth decay.

Among the most studied are genes encoding salivary glycoproteins such as Proline-rich protein 1 (PRH1) and 2 (PRH2), which contribute to the formation of the acquired enamel pellicle and mediate bacterial adhesion. Their allelic variants define specific salivary phenotypes, some of which have been associated with a higher affinity for cariogenic bacteria [2,3,5,17].

Similarly, MUC7, encoding Mucin 7, plays a key role in bacterial aggregation and clearance. Certain polymorphisms in MUC7 may compromise its protective effects [2,17,23].

CA6, which codes for Carbonic anhydrase VI, is essential for buffering capacity and pH regulation in saliva. Reduced enzymatic activity, associated with specific variants, may impair acid neutralisation and increase caries risk [2,3,17,24].

Additionally, Aquaporin-5 (AQP5), a water channel protein gene, regulates salivary flow rate. Alterations in AQP5 expression or function can lead to hyposalivation, diminishing saliva’s protective functions [2,17,23,24].

The antioxidant profile of saliva is influenced by genes such as Nicotinamide phosphoribosyltransferase (NAMPT), involved in NAD (Nicotinamide adenine dinucleotide) biosynthesis and redox balance. While primarily studied for its role in immunity, NAMPT also contributes to salivary antioxidant capacity and may indirectly modulate the oral microbial environment [2,13].

Furthermore, Trefoil factor 2 (TFF2) is expressed in salivary glands and is involved in mucosal protection and repair. Though its role in caries is less well characterised, its inclusion reflects the growing interest in salivary genes that influence mucosal resilience [25].

Collectively, these genes shape both the quantity and quality of saliva. Variations affecting their expression or function may alter the protective potential of the salivary milieu, highlighting their relevance in caries susceptibility and the prospect of genetically informed preventive strategies.

### 2.3. Genes Involved in Oral Immune Response

The oral immune system relies on a fine balance of local defences to prevent caries. Salivary proteins like Lactoperoxidase (LPO) [2,23], β-Defensin 1 (DEFB1) [2,17,23] and lactoferrin (LTF) [2,3,16,17,23] form a first barrier by damaging bacterial membranes, limiting iron availability and modulating immunity; alterations in these genes can weaken antimicrobial action. When bacteria are detected, receptors such as TLR4, CXCR1, and CXCR2 coordinate the immune response by guiding cells to infection sites. Defects here may delay defence and favour dysbiosis [2,17]. In parallel, PRH1 and PRH2 produce proteins that block *S. mutans* adhesion to enamel, though some variants increase the risk [2,3,5,23]. The adaptive response is shaped by HLA-DRB1 and HLA-DQB1, with certain alleles enhancing or limiting microbial colonisation [2,3,5,17,23]. The complement system, involving MBL2 and MASP2, further supports bacterial clearance; mutations may reduce its efficacy [2,3,5,17,23,26]. CTSC and NCF2 [2] aid in destroying pathogens, while IL-1 modulates inflammation, with some variants promoting tissue damage [2,17]. Finally, mucosal integrity is preserved by PLUNC [2,3] and LYZL2 [2,3], and ALOX15 [18,27] may help fine-tune immune regulation. Altogether, genetic variations across these key genes can compromise oral defences and raise susceptibility to caries.

### 2.4. Genes Influencing Taste Perception and Sugar Preference

Taste perception is deeply rooted in genetic variability, with significant implications for dietary habits and oral health. Among the key determinants, genes encoding sweet and bitter taste receptors modulate individual sensitivity to flavours, thereby influencing sugar consumption, a critical behavioural risk factor for dental caries.

The TAS1R2 and TAS1R3 genes code for the subunits of the heterodimeric sweet taste receptor. Polymorphisms such as rs35874116 in TAS1R2 have been associated with reduced sensitivity to sweet compounds. Individuals carrying these variants perceive sugary flavours as less intense, often compensating by increasing sugar intake to achieve the same level of taste satisfaction. This behavioural adaptation leads to more frequent and prolonged sugar exposure in the oral cavity, thereby enhancing the cariogenic potential of the diet [2,3,23,28,29,30]. In parallel, the TAS2R38 gene, which encodes a bitter taste receptor (Taste receptor type 2 member 38), affects sensitivity to bitter substances like PROP (Propylthiouracil). Three genotypic profiles are commonly described based on combinations of the PAV and AVI haplotypes. Individuals with the PAV/PAV genotype are highly sensitive to bitterness, which may lead them to avoid certain vegetables or bitter-tasting foods. In contrast, those with the AVI/AVI genotype exhibit markedly reduced bitter taste perception. This insensitivity often translates into a broader acceptance of highly palatable, energy-dense foods, including sweet products, since the aversive component of bitterness is absent. Interestingly, both extremes of the taste spectrum can lead to higher sugar consumption; the sweet-insensitive seek enhanced taste, while the bitter-insensitive experience fewer dietary deterrents. Heterozygous individuals (PAV/AVI) tend to show intermediate behaviours [3,23,30,31,32].

Beyond taste receptors, the SLC2A2 gene (encoding the Glucose transporter GLUT2) has also been implicated in sweet food preference. Variants such as rs5400 may influence central glucose sensing and reward processing, subtly guiding individuals toward diets richer in simple carbohydrates [2,28,29,31]. Moreover, genes involved in appetite regulation and energy homeostasis, including FTO (Fat mass and obesity-associated gene) and MC4R (Melanocortin-4 receptor), which are involved in appetite regulation, have been linked to a heightened preference for sugary foods through their impact on satiety and reward mechanisms [33,34]. Additionally, GNAT3 (Gustducin), which plays a key role in sweet-taste signal transduction at the level of taste receptor cells, may further contribute to sugar preference by influencing early sensory perception [2,34].

Ultimately, despite different sensory mechanisms, these genetic variations converge towards the same behavioural outcome: an increased preference for sugar. When this predisposition is not recognised or addressed, it contributes to habits that promote dental caries from early childhood. A better understanding of these genetic influences paves the way for personalised dietary advice and targeted prevention in caries management, particularly by identifying individuals at risk who could benefit from early behavioural coaching and nutritional support.

### 2.5. From Candidate Genes to Genome-Wide Approaches

**Figure 2:**
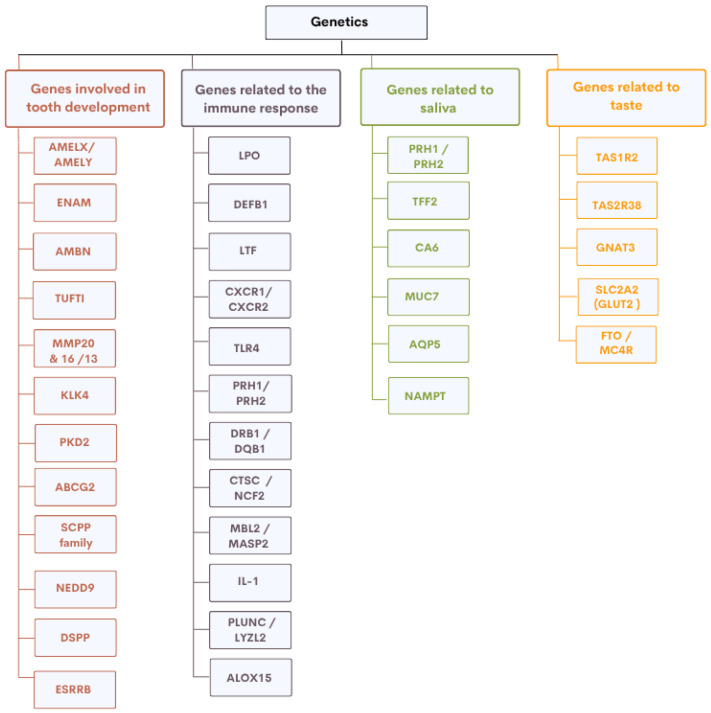
Classification of genes associated with dental caries susceptibility by biological function.

Having reviewed the key genetic factors in tooth development, immunity, saliva, and taste (Figure 2), we next highlight complementary genomic approaches that have uncovered additional, and sometimes unexpected, *loci* associated with susceptibility to dental caries. Two main strategies have been used to investigate the genetic basis of caries: the candidate gene approach and genome-wide association studies (GWAS). The candidate gene approach targets genes with known biological relevance, such as ENAM, which plays a critical role in amelogenesis, or AQP5, involved in salivary secretion. Although often constrained by limited sample sizes, these studies have identified biologically plausible variants.

In contrast, GWAS adopt an agnostic, hypothesis-free approach, scanning the entire genome to identify associations without prior assumptions. This strategy has enabled the development of polygenic risk scores (PRS), which aggregate the effect of hundreds of thousands of common variants. A recent GWAS conducted by Fries et al. in 2024 [4] in a Swedish cohort of 15,460 adults identified ten *loci* with strong predictive contributions to caries risk. These include genes involved in cell signalling (ADCY9—Adenylate cyclase 9, CHRNA3—Cholinergic receptor nicotinic α3), microbiota regulation (FUT2—Fucosyltransferase 2), salivary composition (CA12—Carbonic anhydrase XII), and chromatin structure (HIST1H2BE—Histone H2B type E). Other identified regions included *loci* associated with non-coding or poorly characterised transcripts, such as PITX1-AS1 (PITX1 antisense RNA 1), BAHCC1 (BAH domain containing 1), FAM118A (Family with sequence similarity 118 member A), and C10orf11 (Chromosome 10 open reading frame 11).

Although the functional significance of several of these *loci* in caries pathophysiology remains to be determined, these findings illustrate the complex polygenic architecture underlying dental caries and the value of integrating novel pathways beyond the traditionally studied genes. Nonetheless, the choice of methodological framework significantly affects which genes are identified and how their role is interpreted. While GWAS are powerful for detecting new genetic associations, they have intrinsic limitations. They require large sample sizes to achieve sufficient statistical power and may fail to detect rare variants. Their results can also be confounded by population stratification, particularly in ethnically diverse cohorts. Moreover, GWAS typically explain only a modest fraction of the heritability of caries and do not provide direct evidence of causality.

For this reason, GWAS findings are most informative when combined with complementary methods such as functional genomics, in vitro experiments, and longitudinal cohort studies. Together, these integrative approaches offer the most promising avenue for elucidating the genetic determinants of dental caries and for designing targeted prevention strategies.

## 3. Epigenetics: A Dynamic Modulation

Dental caries has long been recognised as a multifactorial disease involving microbial, dietary, and host-related factors. Recent research has shown that epigenetic mechanisms also play a significant role in caries susceptibility, adding a new layer of complexity without displacing these established contributors. These epigenetic processes enable environmental cues to modulate gene expression without altering the DNA sequence itself, thereby bridging innate genetic predispositions and environmental exposures across the lifespan [5].

DNA methylation, histone modifications, and non-coding RNAs (notably microRNAs) represent the primary epigenetic mechanisms implicated in this dynamic regulation. Recent evidence highlights the relevance of these pathways in shaping caries susceptibility, particularly through prenatal and postnatal influences such as maternal health, nutrition, stress, and early-life microbiota composition.

**Figure 3:**
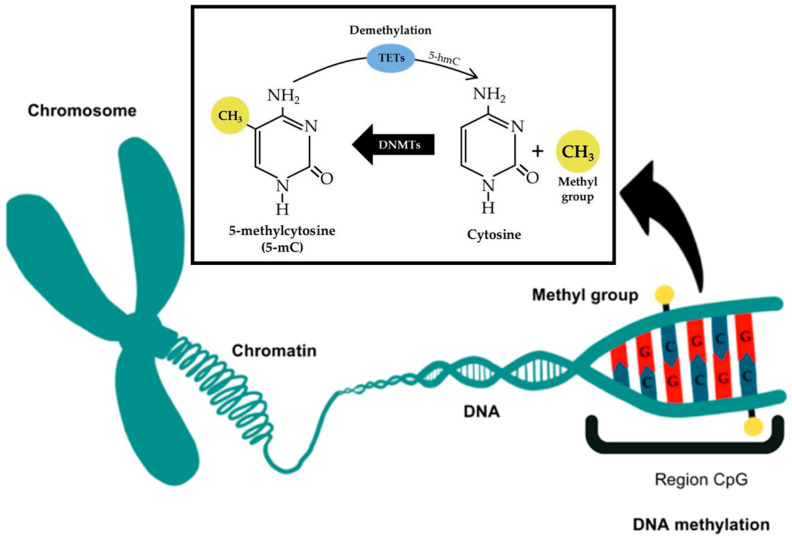
Simplified schematic representation of DNA methylation and demethylation molecular mechanisms, adapted from Valente [36].

**Figure 4:**
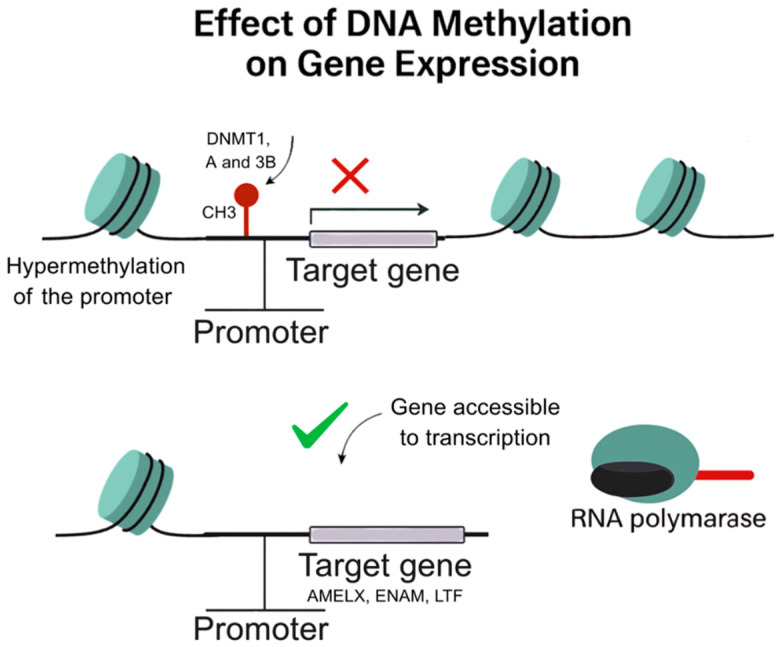
Illustration representing the effect of DNA methylation on gene expression. Promoter hypermethylation by DNMT1, DNMT3A, and DNMT3B leads to transcriptional repression by preventing RNA polymerase binding. In contrast, unmethylated promoters allow transcriptional activation of target genes such as AMELX, ENAM, and LTF.

DNA methylation, one of the most extensively studied epigenetic marks, involves the addition of methyl groups to cytosine residues in CpG dinucleotides (Figure 3). This modification typically represses gene transcription, especially when located in promoter regions (Figure 4) [5]. In the context of dental caries, DNA methylation has emerged as a key mediator of environmental influences during critical developmental windows. The PETS study (Pregnancy and Epigenetics in Tooth Study) exemplifies this perspective. In a cohort of children followed from birth to 6 years, higher levels of DNA methylation in the promoter region of the Early growth response 1 protein (EGR1 gene), encoding a transcription factor involved in cell proliferation, were associated with an increased risk of early childhood caries (ECC). Notably, this methylation pattern was already established at birth in cord blood, suggesting in utero programming of oral health susceptibility [35].

Prenatal environmental exposures can indeed influence the child’s epigenome with long-lasting effects. Maternal obesity, gestational diabetes, smoking, and stress have all been associated with differential methylation patterns in foetal tissues, some of which affect genes related to tooth development, immune regulation or metabolic control. For instance, maternal smoking during pregnancy has been linked to hypermethylation of genes involved in immune responses, potentially compromising neonatal defence mechanisms and favouring the establishment of cariogenic microbiota. Similarly, reduced birth weight, a known risk factor for enamel hypoplasia, has been correlated with altered methylation of growth and differentiation genes, potentially impairing enamel mineralisation and promoting caries development in early childhood [37,38,39].

**Figure 5:**
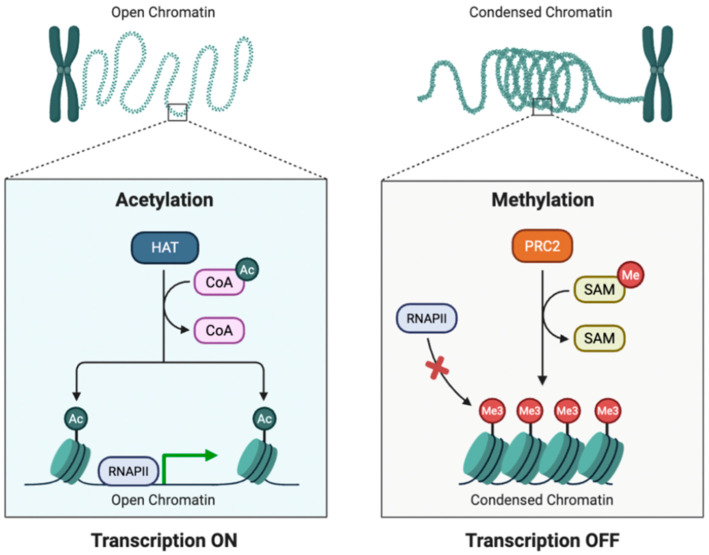
Illustration representing the mechanisms of post-translational histone modifications, including acetylation and methylation. On the left, acetylation by histone acetyltransferases (HAT) adds acetyl groups (Ac) to histone tails using acetyl-CoA (CoA) as a substrate. This modification reduces histone-DNA interactions, resulting in open chromatin structure that is accessible to RNA polymerase II (RNAPII), thereby promoting gene expression (Transcription ON). On the right, methylation is catalyzed by the Polycomb repressive complex 2 (PRC2) using S-adenosyl methionine (SAM) as a methyl donor, leading to tri-methylation (Me3) of histone tails. This promotes a condensed chromatin structure that inhibits RNAPII binding, thereby repressing transcription (Transcription OFF).

Histone modifications add another layer of epigenetic regulation by altering chromatin structure and accessibility of transcriptional machinery (Figure 5). Acetylation of lysine residues, such as H3K9ac, is generally associated with active gene transcription, whereas methylation marks like H3K27me3 are linked to gene silencing [40]. These modifications are regulated by enzymes such as Histone acetyltransferases (HATs), Deacetylases (HDACs), Methyltransferases, and Demethylases. Environmental signals, including microbial products and toxic metals, can modulate the activity of histone-modifying enzymes, thereby altering chromatin structure and gene expression in dental tissues. Arsenic, a widespread environmental pollutant, has been shown to disrupt histone modification patterns, such as reducing histone acetylation and altering histone methylation, which may silence protective genes and promote inflammatory responses in the oral cavity [41,42].

The third key epigenetic mechanism involves non-coding RNAs, including microRNAs (miRNAs) and long non-coding RNAs (lncRNAs). These molecules regulate gene expression after transcription, either by blocking protein production or by degrading messenger RNAs [40]. In the context of dental caries, several miRNAs have been identified as important players. For instance, miR-31 is overexpressed in carious teeth and interferes with Special AT-rich sequence-binding protein 2 (SATB2), which is involved in enamel formation [43], while miR-200c regulates the polarisation of ameloblasts, and its disruption may affect enamel secretion [44]. Furthermore, miR-202 helps control the expression of β-Defensin 1, a key antimicrobial peptide in the mouth, and a variant of this miRNA has been linked to increased caries risk [45]. Finally, miR-146a, which usually calms inflammation, is found decreased in active lesions, allowing pro-inflammatory cytokines like IL-1β and IL-6 to rise [46].

Interestingly, such deregulations in miRNA expression may be driven by environmental stressors, particularly chronic psychosocial stress. It is now well-established that prolonged activation of the hypothalamic–pituitary–adrenal (HPA) axis can lead to sustained cortisol elevation, which in turn induces epigenetic changes. Several studies have shown that stress exposure can alter the expression of stress-responsive miRNAs, such as miR-21 and miR-16, in saliva. These miRNAs are known to influence immune regulation, apoptosis, and inflammation, potentially weakening mucosal defences and increasing susceptibility to microbial colonisation. Although research on stress-responsive lncRNAs is still emerging, early findings suggest they may also play a role in chromatin remodelling and gene expression control in dental tissues. This highlights a possible link between early life stress, epigenetic reprogramming of non-coding RNAs, and an increased risk of dental caries [47].

Taken together, these findings underscore the profound impact of environmental factors on gene regulation through epigenetic pathways. They also reveal how such mechanisms may bridge early-life exposures and long-term oral health outcomes.

In light of this complexity, it becomes essential to adopt a more integrative perspective, one that considers the interplay between genetic predisposition, environmental influences, and epigenetic modulation in the development of dental caries.

## 4. An Integrative View of Dental Caries: Genes, Epigenetics, and Environment

There is clear evidence for a genetic influence on dental caries, as shown by several twin studies. However, this genetic predisposition alone cannot fully explain the variations observed in caries experience. While some studies, such as Bretz et al. (2013), reported high heritability estimates around 64.6%, others found much lower values [3]. For example, Gao et al. (2016) estimated heritability at only 8.7% for early childhood caries [3], and Kuppan et al. [48] reported a heritability of 15% in young Indian twins, highlighting the predominant role of environmental factors such as parental education and feeding habits. These discrepancies shift the focus towards the critical role of the environment, either through direct influences or via epigenetic mechanisms.

**Figure 6:**
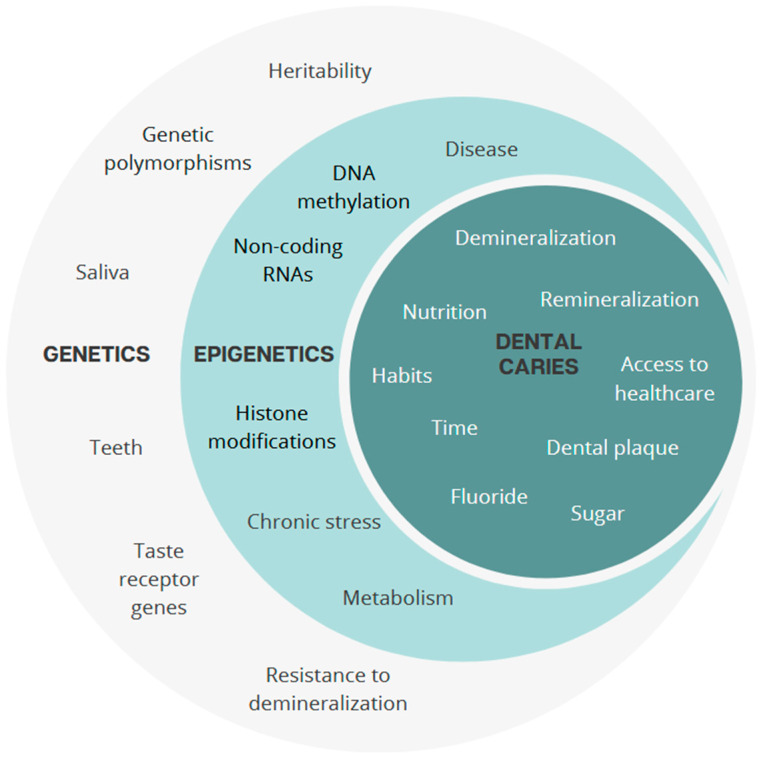
Multifactorial aetiology of dental caries: interaction of genetic variants, epigenetic, and environmental factors.

This has led to a growing consensus around a multifactorial and integrative model in which genetic predispositions interact with environmental exposures and are shaped by epigenetic mechanisms such as DNA methylation, histone modifications and non-coding RNA regulation (Figure 6). Within this framework, the oral microbiota plays a central role as a dynamic mediator between the host genome and the environment. It is no longer considered a static factor, but rather a highly plastic community that influences and is influenced by host biology and lifestyle factors.

This growing consensus around a multifactorial model is reinforced by a series of studies that clarify the role of the oral microbiota not as a passive component, nor merely dictated by host genetics, but as a central, environmentally modulated actor.

Zheng et al. in 2018 [49] provided a striking demonstration of this dynamic by studying monozygotic and dizygotic twin pairs aged 3 to 6 years, discordant for early childhood caries. Using the Human Oral Microbe Identification using Next-Generation Sequencing platform (HOMINGS), which allows high-resolution identification of hundreds of oral bacterial species, the authors found that children with caries harboured significantly higher levels of acidogenic and aciduric species (*S. mutans*, *Scardovia wiggsiae*, *Bifidobacterium longum*) compared to their healthy co-twins. Despite sharing a nearly identical genome and comparable early-life environments, the microbial communities had diverged, suggesting that small variations in diet, hygiene or parental practices can lead to functionally relevant ecological shifts. This study thus supports the idea that the oral microbiota is plastic and modifiable, even within genetically matched individuals, and that environmental modulation predominates beyond the earliest years of life [49].

Zhang et al. in 2015 [50] deepened this understanding by analysing the microbial composition of additional discordant twin pairs. They observed that while dominant species like *Streptococcus* spp. remained present in both twins, the caries-affected siblings exhibited significant differences in the abundance of specific low-prevalence taxa. This finding, enabled by next-generation sequencing, indicates that the shift towards a cariogenic state may not always involve global microbial imbalance, but rather subtle changes in specific niches, highlighting the complexity and ecological specificity of the disease process [50].

Going beyond composition, Gomez et al. in 2017 [51] introduced a functional dimension by performing metatranscriptomic analyses of plaque samples. Rather than merely cataloguing microbial species, they examined the activity of bacterial genes in carious versus healthy sites. They found that caries-associated biofilms expressed higher levels of genes involved in acid production, sugar metabolism, and adhesion functions directly linked to cariogenic potential and heavily influenced by environmental exposures such as frequent sugar intake. These results demonstrate that bacteria contribute to caries not just by being present, but by being metabolically active in response to environmental stimuli. In other words, the oral microbiota possesses its functional genome, which reacts dynamically to the host’s behaviour and diet [51].

Taken together, these studies converge on a key insight: while host genetics and early exposures contribute to shaping the initial microbial landscape, it is the environment, particularly dietary habits and oral hygiene, that modulates both the composition and the activity of the microbiota. The microbiome is not merely a mirror of the host; it is a genetically autonomous, responsive ecosystem that plays an active and evolving role in caries pathogenesis [49,50,51].

These findings highlight the need to move beyond simplified views of inherited risk. Caries susceptibility results from complex interactions between the host, the microbiota, and the environment, shaped by early-life exposures and modulated by epigenetic mechanisms. The oral microbiota acts as a dynamic intermediary, reflecting and responding to behavioural and social contexts.

This understanding supports a more personalised approach to prevention. Rather than applying the same measures to everyone, future strategies could rely on individual microbial or epigenetic profiles to identify those with genetic predisposition. In this light, anti-caries vaccination represents a promising tool to strengthen host defences in those most vulnerable, paving the way for more targeted and effective oral health interventions.

## 5. Towards an Anti-Caries Vaccine: Biological and Practical Challenges

### 5.1. Immunological Principles of Vaccination Against Dental Caries

Dental caries is a multifactorial disease whose main pathogen is *S. mutans*, an acidogenic and aciduric bacterium capable of colonising tooth surfaces for long periods. This ability is based on an arsenal of virulence factors, including biofilm formation, exopolysaccharide synthesis, substrate adhesion, and tolerance to extreme pH variations [1,52]. These functions are made possible by the expression of specific surface proteins such as protein Antigen c (PAc or Cell surface antigen I/II), Glucosyltransferases B, C, and D (GtfB/C/D), Glucan-binding protein B (GbpB), Glutamate-binding protein H (GlnH), and Phosphate-binding lipoprotein (PstS) [52]. The fundamental objective of anti-caries vaccination is to elicit a specific immune response against these key proteins in order to prevent bacterial adhesion, limit the formation of cariogenic biofilm, and inhibit the metabolism of fermentable sugars by *S. mutans* [8,52].

The vaccine approach is therefore based on inducing an adaptive immune response capable of recognising *S. mutans* early on and neutralising it before it colonises the enamel [8,53,54,55]. Unlike conventional vaccines targeting systemic infections, anti-caries vaccines mainly aim to strengthen local immunity in the oral mucosa, while mobilising certain systemic effectors (e.g., IgG in the gingival crevicular fluid). In this context, secretory Immunoglobulin A (sIgA), which is predominant in salivary secretions, breast milk, and gastrointestinal mucosa, plays a key role in local defence. Produced by plasma cells derived from activated B lymphocytes, it is generated from mucosal immunity-inducing centres, such as gut-associated lymphoid tissue (GALT), before migrating to the salivary glands [53,54,55].

Once in the saliva, secretory IgA prevents *S. mutans* from adhering to tooth surfaces and inhibits the formation of bacterial biofilm. This mechanism is crucial in the prevention of caries, as it acts at an early stage of bacterial infection. In addition, Immunoglobulin G (IgG), produced by activated plasma cells in a systemic context, circulates in the blood and gingival fluid. It participates in the neutralisation of bacterial antigens within tissues or during gingival inflammation, thus reinforcing the synergy between local and systemic immunity [53,54,55].

**Figure 7:**
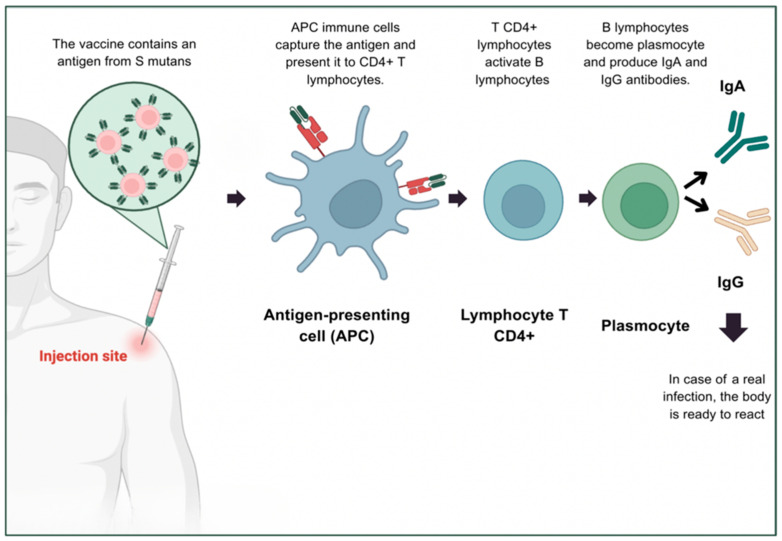
Schematic representation of the immune response initiated by an anti-caries vaccine.

The production of these antibodies is closely regulated by T helper cells (CD4+), particularly T helper 17 (Th17) cells, which are essential for the mucosal response. Their differentiation from naive T cells depends on exposure to a microenvironment rich in cytokines such as IL-6, IL-1β, and IL-23. This cytokine signal directs the cells towards a Th17 phenotype, characterised by the secretion of interleukins IL-17 and IL-22. IL-17 acts as a local alert mediator: it attracts neutrophils, stimulates the production of antimicrobial peptides, and promotes isotype switching to IgA production by B lymphocytes (Figure 7). IL-22, on the other hand, targets epithelial cells to strengthen the mucosal barrier and accelerate tissue repair after inflammation. These cytokines therefore act synergistically to orchestrate a rapid, localised, and effective defence against oral pathogens such as *S. mutans.* These immunological insights provide the foundational framework for the development of innovative vaccine platforms targeting caries prevention [53,54,55].

Considering this, anti-caries vaccine platforms intend to trigger protective immunity based on these dual components: salivary IgA to block bacterial colonisation, and systemic IgG to provide complementary defence. The most widely studied vaccine antigens are PAc proteins, which are involved in bacterial adhesion, Glucosyltransferases (GtfB, GtfC, GtfD), responsible for the production of exopolymers that enable bacterial aggregation, and Glucan-binding proteins (GBP), which anchor bacteria to the biofilm. By targeting these proteins with specific antibodies, the vaccine aims to disrupt the biofilm, reduce bacterial acidogenicity and thus prevent carious lesions [54].

### 5.2. Vaccine Platforms and Antigen Targets

Based on these immunological targets, a wide array of vaccine technologies has been explored. Active immunisation strategies against *S. mutans* aim to induce a specific and lasting immune response by stimulating the host’s adaptive system to recognise and neutralise key antigens involved in the cariogenic process. The ultimate objective is to elicit mucosal sIgA capable of preventing bacterial adhesion and colonisation on enamel surfaces [8].

Among the most explored techniques, recombinant protein-based vaccines have demonstrated strong potential, particularly through the use of key surface proteins such as PAc, GTF, and GbpB. Early formulations using purified bacterial components were progressively replaced by recombinant subunits, offering enhanced antigenic precision, greater safety, and scalable production. A striking illustration of this strategy is the encapsulation of recombinant PAc in ZIF-8 nanoparticles. This subcutaneous vaccine showed improved uptake by dendritic cells and induced a robust T helper 2 (Th2) immune response in mice. In the study by Yu et al. in 2023, the ZIF-8–PAc formulation significantly reduced *S. mutans* colonisation and caries lesion formation [56]. This highlights the advantages of nanoparticle-based adjuvants in injectable vaccine platforms. Despite these promising results, no injectable candidate has yet progressed beyond preliminary clinical trials. Nevertheless, these platforms remain a fundamental model in caries vaccine research and continue to guide the development of safer and more immunogenic antigens and adjuvant systems. However, one of their main limitations is their inability to elicit strong immune responses at the mucosal level, which is essential for effectively preventing a disease that originates on the oral surface [56,57].

This limitation has sparked growing interest in mucosal immunisation strategies, which aim to induce immune responses at the entry site of *S. mutans*. Stimulating the production of sIgA in saliva is a key objective, as these antibodies can block bacterial adhesion to enamel, neutralise virulence factors and inhibit early biofilm formation. To achieve this, mucosal routes of administration such as intranasal, oral, and sublingual are being investigated for their potential to elicit more effective local immune protection compared to systemic injections. A central mechanism behind the efficacy of mucosal vaccines is the activation of Th17 responses, which promote the differentiation of B lymphocytes towards IgA production and enhance epithelial barrier function [8].

This reasoning guided the development of the recombinant KFD2 (Flagellin derivative KFD2)/rPAc vaccine, evaluated by Liu et al. in 2024 [58]. This vaccine combines the PAc adhesin with bacterial Flagellin. Administered intranasally, it induced a robust mucosal response, with high production of salivary IgA and an 83% reduction in caries development, even under conditions of increased virulence. These results demonstrate the efficacy and relevance of a prophylactic mucosal vaccination strategy [58].

Among the mucosal routes studied, the sublingual route has shown potential in inducing systemic and local immune responses while avoiding gastrointestinal degradation of the antigen. In a preclinical study, Ferreira et al. in 2016 [59] evaluated a sublingual vaccine using recombinant PstS, a Phosphate-binding lipoprotein involved in the metabolism and adhesion of *S. mutans*. When administered to BALB/c mice, the vaccine, particularly when combined with the mucosal adjuvant LTK4R (non-toxic derivative of the *Escherichia coli* labile toxin), elicited strong systemic IgG and mucosal IgA responses. The immune response was dominated by a Th2-type cytokine profile, characterised by high levels of IL-6 and IgG1, reflecting a humoral response geared towards antibody production. Following this experiment, antibodies from vaccinated animals inhibited bacterial adhesion in naïve mice, confirming both the immunogenicity and functional targeting of PstS [59].

Building on previous work, Pereira et al. in 2022 [10] investigated another ABC transporter component, GlnH, a Glutamate-binding protein involved in acid tolerance and essential metabolism in *S. mutans*. The recombinant antigen, produced in *Bacillus subtilis*, was administered sublingually, with or without the adjuvant LTK63 (mutant *E. coli* Heat-labile toxin derivative (Ser63 → Lys mutant)). Similarly to the PstS vaccine, the GlnH antigen induced strong IgG responses and significantly reduced oral colonisation by *S. mutans*, despite the absence of a significant mucosal IgA response. These findings support the potential of ABC transporter proteins as effective targets for mucosal vaccines against dental caries [10].

Beyond microbial expression systems, plant-derived recombinant vaccines have emerged as an innovative platform in mucosal immunisation. This strategy involves the genetic transformation of edible plants like tobacco or lettuce to produce immunogenic antigens such as PAc, thereby transforming plant tissues into antigen reservoirs. Administered orally or nasally in the form of powdered plant material, this platform offers economic, logistical, and safety advantages [60]. While direct application in caries prevention is still pending, the success of similar vaccines, such as the Covifenz^®^ COVID-19 (Coronavirus disease 2019) vaccine based on *Nicotiana benthamiana*, demonstrates the feasibility of this model. Its advantages include thermostability, ease of distribution, and acceptability in paediatric populations, making it a compelling prospect for large-scale deployment in low-resource settings [61].

DNA-based vaccines offer an alternative to protein subunits by delivering genetic blueprints for antigen production directly into host cells. This approach stimulates both humoral and cellular responses following endogenous synthesis and presentation of *S. mutans* antigens. In a notable example, Jiang et al. in 2017 [62] developed a dual-promoter DNA vaccine encoding SBR and GBR fragments of PAc and GTF-I, delivered orally via live attenuated *Salmonella typhimurium*. This design enhanced antigen expression in both bacterial and host cells, resulting in elevated salivary IgA and serum IgG levels, along with a 99% reduction in bacterial colonisation in mice. These findings demonstrate that combining DNA vaccines with mucosal bacterial vectors significantly improves immune priming [62]. Another study by Yan et al. further explored this avenue by using a plasmid encoding a conserved region of PAc, co-administered intranasally with chemokine adjuvants CCL17 and CCL19. Vaccinated rats displayed enhanced IgA levels in saliva and reduced carious lesions, validating the mucosal potential of DNA immunisation [63].

A particularly innovative approach, still under theoretical development, involves live attenuated viral vectors. The use of cold-adapted influenza virus (CAIV) as a delivery platform for *S. mutans* antigens has been proposed as a promising avenue. This strategy would involve inserting antigenic epitopes such as PAc, GTF or GBP into the CAIV genome, leveraging its proven safety, large-scale manufacturability, and intranasal administration potential. As outlined by Yang et al. in 2019 [11], this vector could induce durable mucosal memory and sIgA responses in children before oral colonisation occurs. Though no experimental data are available yet, the regulatory acceptance and immunogenicity of CAIV in other infectious contexts make it a strong candidate for future development in paediatric dental immunisation [11].

Passive immunisation, on the other hand, relies on the direct administration of preformed antibodies capable of neutralising a pathogen without requiring the host’s immune system to mature. This strategy, which has already been used successfully against various viral and bacterial infections, offers immediate and targeted protection. It is an interesting alternative in the context of dental caries, particularly in young children or immunocompromised individuals, in whom it is sometimes difficult to induce a robust vaccine response [8,54].

The production of recombinant immunoglobulins in transgenic plants represents a major advance in the development of effective, safe, and accessible passive immunotherapies. Genetically modified plants can produce large quantities of *S. mutans*-specific IgA, which can neutralise the pathogen directly in the mouth. These antibodies, expressed by plants such as tobacco or lettuce, retain their activity after purification and can be formulated into gels, sprays or oral tablets. Vasilev et al. demonstrated the feasibility of this strategy by producing functional mucosal IgA through stable transformation or transient expression via viral vectors, while highlighting the advantages in terms of cost, safety, and stability [60].

This approach was anticipated by the pioneering study by Ma (1998) [64], which remains the only experimental demonstration in humans to date of the efficacy of secretory IgA antibodies produced in transgenic plants against *S. mutans*. In this study, IgA specific to the AgI/II (PAc) antigen was produced in tobacco plants, purified, and then administered locally to volunteers. The results showed transient inhibition of biofilm formation, although the antibodies did not persist in the oral cavity for more than three days [64].

In parallel with this plant-based strategy, other biotechnological approaches have been developed, including the production of human Fragment antigen-binding (Fab) fragments using phage display technology. Fab fragments, which contain only the variable region of the antibody responsible for antigen recognition, offer excellent tissue diffusion and low immunogenicity. Alam et al. in 2018 [9] produced Fab directed against *S. mutans* and *S. sobrinus*, selected for their high affinity via a phage library expressing antibody fragments. These Fab showed marked inhibition of biofilm formation in vitro. When applied topically to infected rats fed a cariogenic diet, they significantly reduced the incidence of carious lesions. Micro-CT imaging revealed a fivefold reduction in the number of lesions in the treated groups, highlighting the potential of this targeted and innovative strategy.

Despite their proven efficacy, passive immunisation strategies suffer from certain limitations, notably the short half-life of antibodies in saliva, requiring repeated applications. Recent research aims to improve oral retention through bioadhesive formulations or to prolong the half-life of antibodies through molecular engineering. Approaches combining passive immunisation, probiotics, fluorides or dietary modifications are also being studied to optimise protection against caries [12].

### 5.3. Feasibility and Limitations of Large-Scale Application

The development of vaccines against dental caries, particularly those targeting *S. mutans*, represents a promising public health strategy, especially for populations with limited access to dental care. While young children remain the ideal target for early intervention, given the timing of primary colonisation and the risk of early caries, no paediatric clinical trials have yet confirmed their safety or efficacy [1]. Other groups, such as the elderly, persons with disabilities, and socially vulnerable communities, could also benefit from vaccination strategies that offer passive protection and reduce the need for invasive care [65].

Beyond the identification of clinical target groups, vaccination also offers the potential to reduce social inequalities in oral health, particularly if made universally accessible through public health programmes. By providing immune protection regardless of socioeconomic status or access to dental care, such an approach could promote equity [1]. In economic terms, an effective anti-caries vaccine could lower both the direct costs of treatment and the broader societal costs linked to absenteeism and reduced productivity. However, no formal economic model has yet confirmed these expected benefits [66].

In addition to population-based and economic considerations, interindividual biological variability remains a critical factor in the development of anti-caries vaccines. Genetic differences, particularly in HLA and TLR genes, can influence how antigens are recognised and how the immune system responds, leading to differences in vaccine efficacy and side effects. This highlights the potential value of genetic and epigenetic profiling to guide future strategies either by identifying individuals most likely to benefit from vaccination or by adapting vaccine design to better match host immune characteristics [67,68].

Host genetics influences the composition of the oral microbiota, which in turn plays a key role in regulating mucosal immune responses. For example, certain microbial profiles can promote the production of protective IgA antibodies or influence how T cells respond to vaccination [67]. This means that the effectiveness of mucosal vaccines may vary depending on each person’s microbiota. These insights open the door to personalised vaccination strategies, but their implementation would require complex testing and raise ethical and logistical challenges that currently prevent their large-scale use.

Despite these challenges, experimental evidence supports the scientific feasibility of anti-caries vaccination. In a recent narrative review, Contreras et al. [69] identified eleven key studies evaluating vaccine candidates targeting *S. mutans*, most of which were conducted in vivo in animal models. These studies consistently demonstrated that several formulations, including recombinant, DNA-based, plant-derived, and vector-based vaccines, were able to induce local or systemic immune responses, notably through the production of salivary IgA, while significantly reducing bacterial load and caries severity [69].

The review also highlighted important advances in vaccine design, including the use of innovative adjuvants such as Chitosan, Flagellin, and LTK4R to enhance mucosal immunogenicity. However, Contreras et al. [69] emphasised that the vast majority of the available data remain preclinical. To date, the only documented human study conducted by Ma et al. in 1998 reported a humoral immune response following experimental nasal immunisation in adults, but no follow-up clinical trials have been reported since Ma et al.’s 1998 human pilot study [64].

These findings confirm that anti-caries vaccination is scientifically promising, but they also underscore the need to validate immune durability, mucosal vector safety, clinical efficacy, and cost-effectiveness in real-world settings before widespread application can be considered.

## 6. Conclusions

Our knowledge of dental caries has evolved considerably in recent decades, moving beyond a purely behavioural or microbiological perspective to incorporate a multifactorial approach that combines genetics, the environment and host biology. This work shows that while some individuals have a genetic predisposition to caries involving polymorphisms that influence enamel quality, saliva composition, mucosal immune response or even taste preferences for sugar, this predisposition is strongly modulated by epigenetic and environmental factors.

These environmental factors are numerous and act from the earliest stages of life. They include early and regular consumption of fermentable sugars, low exposure to fluoride, poor oral hygiene, and unfavourable socioeconomic conditions that limit access to care and prevention. Perinatal factors, such as mode of delivery, infant feeding (breastfeeding vs. bottle feeding), and exposure to oral microbiota disruptors (antibiotics, passive smoking), can also influence the maturation of the immune system and microbiota, thereby contributing to individual vulnerability to caries. These influences can act via epigenetic mechanisms (DNA methylation, microRNA), interfering with the expression of genes related to immunity, mineralisation, and microbiota regulation.

This complex interaction between early environmental exposures, host biology, and epigenetic regulation not only shapes individual susceptibility to caries but also highlights the limitations of conventional preventive strategies. Furthermore, a growing body of evidence suggests that certain genetic determinants, such as dental topographical vulnerability and sex-related differences in immune response, may influence both caries risk and responses to interventions. In this context, innovative approaches such as vaccination appear to be promising avenues for strengthening host defences and disrupting cariogenic mechanisms, while paving the way for sex-specific prevention strategies.

Vaccination against *S. mutans* appears to be an innovative avenue with great potential. The preclinical studies summarised in this review confirm the biological feasibility of several vaccine platforms (recombinant, DNA, plant-based, attenuated vectors) capable of significantly reducing bacterial colonisation and the severity of carious lesions in animals. However, their translation into human clinical practice remains subject to several crucial challenges: the durability of the mucosal response, the tolerance of mucosal vectors, efficacy in real-world conditions, and the influence of genetic variability, including gender, on the immune response. In addition, there is a need for rigorous cost–benefit analyses, particularly in low-resource settings.

In summary, this review highlights the need to address dental caries from an integrated, personalised, and preventive perspective, considering the complex interactions between the genome, environment, immunity, microbiota, and social factors. The future of dental caries prevention lies in an integrated approach based on genetic data, immune mechanisms, and advances in vaccination.
